# Stimulus number, duration and intensity encoding in randomly connected attractor networks with synaptic depression

**DOI:** 10.3389/fncom.2013.00059

**Published:** 2013-05-09

**Authors:** Paul Miller

**Affiliations:** Volen National Center for Complex Systems and Department of Biology, Brandeis UniversityWaltham, MA, USA

**Keywords:** short-term plasticity, dynamic synapses, attractor networks, short-term memory, distributed coding, high-dimensional representation

## Abstract

Randomly connected recurrent networks of excitatory groups of neurons can possess a multitude of attractor states. When the internal excitatory synapses of these networks are depressing, the attractor states can be destabilized with increasing input. This leads to an itinerancy, where with either repeated transient stimuli, or increasing duration of a single stimulus, the network activity advances through sequences of attractor states. We find that the resulting network state, which persists beyond stimulus offset, can encode the number of stimuli presented via a distributed representation of neural activity with non-monotonic tuning curves for most neurons. Increased duration of a single stimulus is encoded via different distributed representations, so unlike an integrator, the network distinguishes separate successive presentations of a short stimulus from a single presentation of a longer stimulus with equal total duration. Moreover, different amplitudes of stimulus cause new, distinct activity patterns, such that changes in stimulus number, duration and amplitude can be distinguished from each other. These properties of the network depend on dynamic depressing synapses, as they disappear if synapses are static. Thus, short-term synaptic depression allows a network to store separately the different dynamic properties of a spatially constant stimulus.

## Introduction

Circuits of reciprocally connected neurons have been long considered as a basis for the maintenance of persistent activity (Lorente de Nó, 1933). Such persistent neuronal firing that continues for many seconds after a transient input can represent a short-term memory of prior stimuli (Funahashi et al., 1991). Indeed, Hebb's famous postulate (Hebb, 1949) that causally correlated firing of connected neurons could lead to a strengthening of the connection, was based on the suggestion that the correlated firing would be maintained in a recurrently connected cell assembly beyond the time of a transient stimulus (Hebb, 1949). Since then, analytic and computational models have demonstrated the ability of such recurrent networks to produce multiple discrete attractor states (Brunel and Nadal, 1998), as in Hopfield networks (Hopfield, 1982, 1984), or to be capable of integration over time via a marginally stable network, often termed a line attractor (Zhang, 1996; Compte et al., 2000). Much of the work on these systems has assumed either static synapses, or considered changes in synaptic strength via long-term plasticity occurring on a much slower timescale than the dynamics of neuronal responses. Here we add some new results pertaining to the less well-studied effects of short-term plasticity—changes in synaptic strength that arise on a timescale of seconds, the same timescale as that of persistent activity—within recurrent discrete attractor networks.

The two long-established forms of short-term synaptic plasticity affect all synapses of the presynaptic cell according to its train of action potentials. Synaptic depression refers to a reduced synaptic efficacy in the few hundreds of milliseconds following a presynaptic spike, effectively weakening connections strengths as presynaptic firing rate increases (Markram and Tsodyks, 1996; Abbott et al., 1997). Such weakening of efficacy of the most active connections has an unavoidable destabilizing effect on any network state that depends on those active connections for its persistence. Synaptic facilitation is the opposite effect—a temporary enhancement of synaptic efficacy in the few hundreds of milliseconds following each spike (Markram et al., 1998), effectively strengthening connections to post-synaptic cells as presynaptic firing rate increases.

More recently described and information-theoretically more powerful than depression or facilitation, is an associative form of short-term plasticity (A-STP), which depends on both pre- and post-synaptic activity (Erickson et al., 2010). A-STP produces a temporary enhancement of synaptic efficacy between neurons after a short period of strong coactivity. Being a form of positive feedback, A-STP, like facilitation, is likely to stabilize states of persistent activity, but may have the added benefit of maintaining sequences of persistent firing states (Miller and Wingfield, 2010).

In this paper, we focus on short-term synaptic depression in randomly connected networks of discrete attractors (Rigotti et al., 2010). The attractors are formed by coupling multiple groups of neurons, each group rendered bistable through recurrent excitation. The destabilization of discrete attractor states by short-term synaptic depression produces a rich repertoire of network responses, allowing it to encode and store multiple stimulus features.

Short-term depression arises from vesicle depletion (von Gersdorff and Matthews, 1997), which leads to a maximum, saturating rate of synaptic transmission—dependent on the rate of vesicle recycling. The temporary weakening of connection strengths from active cells tends to reduce the stability of active recurrent cell-groups. This can lead to more dynamic or itinerant activity states in recurrent networks. Here we show that in a network of randomly coupled cell-groups, the itinerancy produced by synaptic depression can cause the network to reach a state that depends on any of stimulus intensity, or stimulus duration or the number of successive identical stimuli presented. In the latter case, neurons can be tuned to a specific number of inputs, similarly to those recorded *in vivo*.

Counting of stimuli can be achieved without dynamic synapses in a network behaving as an integrator. Indeed, appropriate feedforward connections from an integrator can produce numerosity-tuned neurons (Verguts and Fias, 2004), with similar tuning curves to those found *in vivo* (Nieder and Miller, 2003; Tudusciuc and Nieder, 2007; Merten and Nieder, 2009; Nieder, 2013). However, an integrator, whether it arises from a finely tuned network with a continuous, line attractor (Seung et al., 2000; Miller et al., 2003; Machens et al., 2005), or more robustly from a series of discrete attractor states (Koulakov et al., 2002; Goldman et al., 2003), is not ideal as the input to a counter. While a perfect integrator does indeed produce distinct responses to successive identical stimuli, it conflates both amplitude and duration of the stimulus, with the number of stimuli, into a single response that only depends on the produce of these three quantities. Thus, an integrator's response to two stimuli of a given magnitude and duration is identical to that of a single stimulus with either twice the magnitude or twice the duration. Any non-linearities would remove such perfect scaling [which is essential in situations requiring perfect integration, such as from velocity to position (Zhang, 1996; Samsonovich and McNaughton, 1997; Song and Wang, 2005)] but would not remove the conflation of stimulus features, since an integrator's activity is confined to a one-dimensional surface—input amplitude, duration and number produce shifts along the same one-dimensional line. Thus, for an integrator to act as a counter, its inputs must be first scaled to a fixed duration and amplitude by upstream sensory processing.

Here we test whether any advantage over the integrator is offered by the high-dimensional space of attractor states produced by randomly connected bistable groups of neurons (Rigotti et al., 2010). In a group of cells with recurrent excitatory connections, the excitability of the cell-group—its ability to become rapidly active in response to input—increases with the effect strength of the internal connections. In a network with many such cell-groups, if they are predominantly coupled by cross-inhibition, those cell-groups most excited by the stimulus and activated most quickly, can suppress activity of other cell-groups. Short-term synaptic depression reduces the effective connection strengths between coactive neurons compared to those between quiescent neurons. Since the amplitude of synaptic depression is firing-rate dependent, and since internal randomness in the network causes cell-groups to respond with different amplitude-dependences of their firing rates, stimuli of different amplitudes are likely to affect the network differently. Moreover, dynamical synapses cause the network's response to depend on the temporal profile of stimuli, not just its temporal integration, so that two spaced stimuli could produce a different response from a single stimulus of twice the duration.

Therefore, we will vary three stimulus properties—number, duration and amplitude—both individually and together, to assess whether a randomly connected network with dynamic synapses, unlike an integrator, can dissociate these features. We first assess whether, when a stimulus is repeated, cell-groups active to its first presentation can be replaced by other active cell-groups during its second and later presentations. We then uncover how this process, in a randomly connected sparse recurrent network, depends on different qualities of the stimulus, such as its duration and intensity. Finally, we show these qualities interact with the number of stimuli in a non-trivial manner, often producing unique patterns of persistent activity as a function of number, duration and intensity of preceding stimuli.

## Methods

### Firing rate model with depressing synapses

To model the effects of synaptic depression in a network of coupled cells, we employ a firing rate model, which treats the mean input current, *I*_*i*_(*t*), the mean firing rate *r*_*i*_(*t*), the mean depression variable, *D*_*i*_(*t*) and the mean synaptic output, *S*_*i*_(*t*), of individual groups of neurons, labeled *i*, as continuous, time-dependent quantities. The formulation is appropriate for cells with Poisson spike statistics, as at fixed firing rates the depression variable and synaptic outputs approach the steady state values produced by Poisson spike trains, though with appropriate rate-dependent modifications to the effective time constants. Thus, the dynamics of the system is described by a set of coupled first order differential equations. The firing rate depends upon its input current according to a sigmoidal f–I curve, as:

(1) $$\tau_{r}\frac{dr_{i}}{dt}=-r_{i}(t)+\frac{r_{i}^{\text{max}}}{\exp\left\{\left[\Theta_{i}-I_{i}(t)\right]/\Delta_{i}\right\}}$$

where τ_*r*_ = 10 ms is the time constant for, *r*^max^_*i*_ is the maximum firing rate of that cell-group, Θ_*i*_ is the threshold, namely the level of input current required for half-maximal firing and Δ_*i*_ determines (with *r*^max^_*i*_) the slope of the f–I curve.

The depression variable follows:

(2) $$\tau_{Di}\frac{dD_{i}}{dt}=1-D_{i}(t)-p_{0}r_{i}(t)\tau_{Di}D_{i}(t)$$

where *p*_0_ is the fraction of docked vesicles released per spike and τ_*Di*_ is the recovery time to regain maximum transmission. Equation 2 is chosen so as to reach the steady state value produced by a Poisson spike train (Dayan and Abbott, 2001) of rate *r*_*i*_:

(3) $$D^{ss}(r_{i})=\frac{1}{1+p_{0}r_{i}\tau_{Di}},$$

if the rate were fixed, assuming each presynaptic spike at time *t*_*s*_ causes a reduction in the depression variable, *D*_*i*_(*t*^+^_*s*_) = *D*_*i*_(*t*^−^_*s*_)(1 − *p*_0_), due to loss of a proportion, *p*_0_, of docked vesicles.

The synaptic gating variable follows:

(4) $$\tau_{s}\frac{ds_{i}}{dt}=-s_{i}(t)+\tilde{\alpha }p_{0}r_{i}(t)\tau_{s}D_{i}(t)\left[1-s_{i}(t)\right]$$

where τ_*s*_ is the synaptic time constant for decay of *s*_*i*_ to zero in the absence of synaptic transmission and $\tilde{\alpha }$ is the fraction of open receptors bound by maximal vesicle release—that is, the fractional increase in *s* for a given presynaptic spike at time *t*_*s*_ is $\tilde{\alpha }p_{0}D_{i}(t_{s}^{-})\left[1-s_{i}(t_{s}^{-})\right]$. Equation (3) reaches the steady state value for *s*_*i*_ produced by a Poisson train of releases with fixed *D*_*i*_, at a rate *r*_*i*_:

(5) $$\tau_{s}S^{ss}\left(r_{i},\;D_{i}\right)=\frac{\tilde{\alpha }p_{0}D_{i}r_{i}\tau_{s}}{1+\tilde{\alpha }p_{0}D_{i}r_{i}\tau_{s}}.$$

The connectivity matrix, *W*_*i* → *j*_ describes the connection strengths from each cell-group *i* to cell-group *j*, so determines the input current to a cell-group *j* via:

(6) $$I_{j}(t)=\sum_{i}s_{i}(t)W_{i\to j}+I_{j}^{\text{app}}(t)+\sigma \eta (t)$$

where *I*^app^_*j*_(*t*) is the stimulus-dependent external, applied current to cell-group *j* and η(*t*) is a white noise term which contributes fluctuations to each cell-groups current, with a standard deviation σ.

Full details of the simulation parameters are given in Tables 1 and 2.

Table 1**Components of the network simulations (Nordlie et al., 2009)**.

**Table:** 

**A. Model summary**	
Populations	100 excitatory (E), 1 inhibitory (I)
Connectivity	E-to-E: all-to-all with random strength; high self-excitation
Neuron model	Firing rate model with sigmoidal f–I curve
Synapse model	Single exponential with depression or facilitation
Plasticity	No long-term plasticity
Input	Fixed current pulses to all populations
Measurements	Persistent firing rates after current offset

**Table:** 

**B. Populations**		
**Name**	**Elements**	**Size**
Excitatory (E)	Firing-rate model	1 for each of 100 model cell-populations
Inhibitory (I)	Firing-rate model	1 for the single cell-population

**Table:** 

**C. Connectivity**			
**Name**	**Source**	**Target**	**Pattern**
EE (S)	E	Same E	Fixed at WEEs
EE (X)	E	All other E	All-to-all with weight a random iid in [0, 2WEEx]
EI	E	I	Fixed, constant at WEI
IE	I	E	Fixed, constant to all at WIE
II	I	I	Not Present

**Table:** 

D. Neuron and synapse model	
Name	Firing rate model with dynamical synapses
Type	Dynamic leaky integrate-and-fire, exponential conductance input
Input current	$I_{j}(t)=\sum_{i}s_{i}(t)W_{i\to j}+I_{j}^{app}(t)+\sigma \eta (t)$
Firing rate	$\tau_{r}\frac{dr_{i}}{dt}=-r_{i}(t)+\frac{r_{i}^{\max}}{\exp\left\{\left[\Theta_{i}-I_{i}(t)\right]/\Delta_{i}\right\}}$
Depression variable	$\tau_{Di}\frac{dD_{i}}{dt}=1-D_{i}(t)+p_{0}r_{i}(t)\tau_{Di}D_{i}(t)$
Synaptic transmission	$\tau_{s}\frac{ds_{i}}{dt}=-s_{i}(t)+\tilde{\alpha }p_{0}r_{i}(t)\tau_{s}D_{i}(t)\left[1-s_{i}(t)\right]$

**Table:** 

**E. Plasticity**
No long-term plasticity present

**Table:** 

**F. Input**	
**Type**	**Description**
Applied current	Transient pulses of fixed current with number of pulses, amplitude of pulse and length of pulse varied across simulations. Current is identical to all excitatory populations and scaled by a constant factor to the inhibitory population

**Table:** 

**G. Measurements**	
Firing rates vectors	Mean rate per cell 750–1500 ms after stimulus onset
Correlations	Correlation between firing vectors for different stimuli
Confusability	Proportion of trials that response to a test stimulus is closer to the mean response produced by a target stimulus than to the mean response of any other target stimulus

Table 2**Network simulation model parameters**.

**Table:** 

**A. Firing rate model and input current**								
**Population**	**τ_*r*_**	**r^max^_*i*_**	**Θ_*i*_**	**Δ_*i*_**	σ
Excitatory (E)	0.01 s	100 Hz	[6.3–6.5]	1	0.002, 0.005
Inhibitory (I)	0.01 s	200 Hz	12	3	0.002, 0.005

**Table:** 

**B. Depression and synaptic transmission**								
**Connection**	**τ_*D*_**	***p*_0_**	**τ_*s*_**	$\tilde{\alpha }$
EE (S), EE (X), EI	0.5 s	1	0.05 s	1
IE	0.5 s	0.1	0.005 s	1

**Table:** 

**C. Connection strengths**								
**Connection**	***W*^0^_*EE*_**	***W*^*X*^_*EE*_**	***W*_*EI*_**	***W*_*IE*_**
Value	85	[0–0.4]	2.5	−300

**Table:** 

**D. Stimulus values**								
**Property**	***N*_max_**	***T***	***I*_0_**	**Interval**
Value	10 (8, 6, 1)	0.1 s (0.01 s–1 s)	2 (0.5–3)	1.5 s (2 s)

### Network properties, stimulation protocols and measurements

Our main results were achieved with a network of *N*_*E*_ = 100 excitatory cell-groups and a single inhibitory cell-group, though we tested the effects of using from *N*_*E*_ = 20 to *N*_*E*_ = 400 excitatory cell-groups. The dominant connections within the network were produced by strong self-excitation within each excitatory cell-group and strong cross-inhibition between all excitatory cell-groups via the inhibitory cell-group. The cell-groups were further coupled by all-to-all excitatory connections, with connection strength chosen randomly from a uniform distribution between zero and the maximum value. Such random cross-connections, even in sum, produced a weaker excitatory input than the within-group connection.

More specifically, the connection matrix, *W*_*i* → *j*_ (Equation 6) comprised four types of connection: fixed strength excitatory connections within an excitatory cell-group (*W*_*i* → *i*_ = *W*^0^_*EE*_ for 1 ≤ *i* ≤ *N*_*E*_); random strength excitatory connections between excitatory cell-groups (*W*_*i* → *j*_ = ξ_*ij*_*W*^*X*^_*EE*_/(*N*_*E*_ − 1), if *i* ≠ *j* and 1 ≤ *i*, *j* ≤ *N*_*E*_) and η_*ij*_ is a random number selected from a uniform distribution (0 < ξ_*ij*_ < 1); fixed strength excitatory connections to the inhibitory cell-group (*W*_*i* → *j*_ = *W*_*EI*_/(*N*_*E*_ − 1) if 1 ≤ *i* ≤ *N*_*E*_ and *j* = *N*_*E*_ + 1); and fixed strength inhibitory connections to each excitatory cell-group (*W*_*i* → *j*_ = *W*_*IE*_ if *i* = *N*_*E*_ +1 and 1 ≤ *j* ≤ *N*_*E*_). Values of these parameters are given in Table 2. Different versions of a network with the same parameters were generated by selecting a new set of random excitatory cross-connections through a new generation of the random matrix, ξ_*ij*_. In contrast, repeated trials with the same network were produced with a fixed connection matrix, *W*_*i* → *j*_, but with a new instantiation of trial-specific random noise in the simulation, via η(*t*) (Equation 6).

Stimuli were trains of transient current pulses, with each pulse producing the same current input to all excitatory cell-groups, as well as an input to the inhibitory cell-group. Depending on the protocol, current pulses ranged in number from 1 to 10, in duration from 10 ms to 1 s and in amplitude from 0.5 to 3 (in units where the firing threshold was in the range 6.3–6.5 for excitatory cells). Current pulses were delivered every 1.5 s in all protocols, except for those with varying stimulus duration, in which case delivery was every 2 s. While these current pulses could evoke immense changes in network activity, even the strongest inputs contributed only a small fraction of the total input to any cell-group, as the network is dominated by feedback within the circuit.

Mean network activity was calculated in all cases from at least 100 ms after stimulus offset until the onset of the subsequent stimulus. In the standard protocol, with a stimulus of 250 ms, rates of each cell were averaged from 375 to 1500 ms from stimulus onset (i.e., 125–1250 ms from stimulus offset) to determine the stimulus responses used in later analyses.

### Confusability matrix

To calculate a confusability matrix, we first simulated a set of 10 different random trials of the same network with different instances of noise via η(*t*) (Equation 6). We used these initial trials to obtain the mean response in the delay period following each stimulus number or stimulus type, and defined these mean responses as the “target response.” We then simulated a new set of 10 different random trials (“test trials”) of the same network, for each test trial assessing which target response the delay activity most closely resembled. The confusability matrix gives the fraction of test trials, for which the response to one stimulus type and number most closely resembles the “target response” of a given stimulus type and number.

### Weber scaling

To test for Weber's law, we produced 10 distinct networks, with 25 target trials and 25 test trials in each network. Importantly, across trials we allowed the level of noise to vary randomly, in this case according to a uniform distribution over the range 0.0015 < σ < 0.0075. For each network, for a given test stimulus number, we calculated the mean and standard deviation of the target stimulus number the delayed activity most closely resembled. We then plot the mean standard deviation across networks versus the mean target reached in Figure 2C.

## Results

### Numerosity

**Figure 1:**
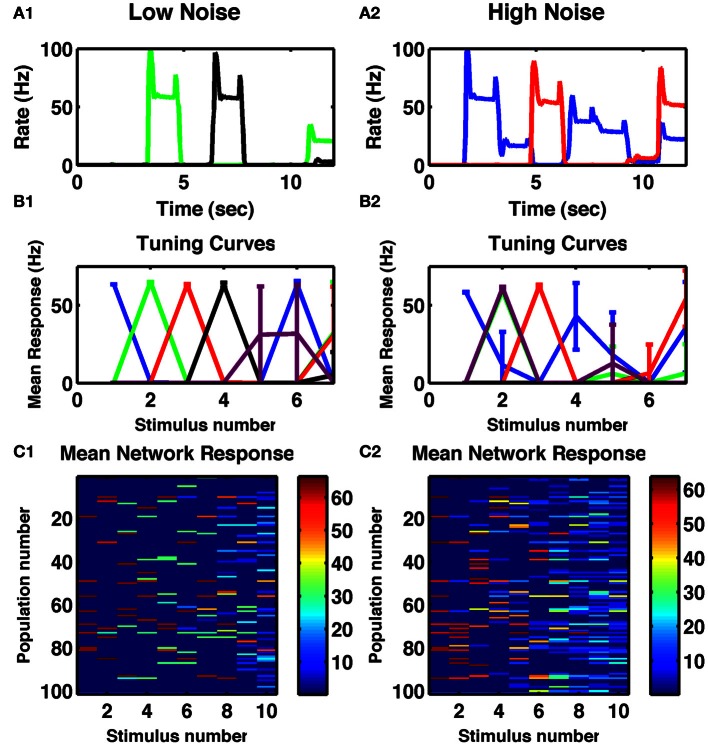
**Cell-group activity is tuned to the number of repeated stimuli. (A1)** Responses of two different cells to a series of 250 ms constant inputs repeated every 1.5 s. **(A2)** Responses of two other cells to the same sequence with increased noise in the network. **(B1,B2)** Tuning curves of specific cell-groups in the network, each color represents a different cell, whose firing rate is plotted as a function of the number of successive identical stimuli. Colors matching those in **(A1,A2)** indicate the same cell. **(C1,C2)** Mean delay activity of all cell-groups to a train of ten identical stimuli, with color indicating firing rate. **(A1,B1,C1)** Internal noise, σ = 0.002. **(A2,B2,C2)** Increased internal noise, σ = 0.005. All panels: Mean responses averaged across ten trials of a single network, with error bars indicating standard deviations.

Numerosity is the ability of a circuit to represent the number of transient stimuli. In the first task, we simply applied, repeatedly, a constant transient stimulus current to all cell-groups and assessed how reliably the resultant activity depended on the number of stimuli to date. Given appropriate parameters—in particular such that recurrent self-excitation within cell-groups was sufficient to maintain activity beyond the time of the transient stimulus (Figure 1A), but not so strong that it could not be suppressed by cross-inhibition arising from later activity in other cell-groups—the network could switch through stable, distributed activity states as shown in Figure 1. Moreover, when averaging single-cell responses during the delays between stimuli across 10 trials, many cells were tuned to individual numbers of stimuli (Figure 1B1). With increased noise, the observed tuning was broader for neurons selective to higher numbers (Figure 1B2). Similar tuning is seen in the neural activity of numerosity-selective neurons in primates (Nieder and Miller, 2003; Tudusciuc and Nieder, 2007, 2009), neurons which also respond to a temporal sequence of discrete stimuli (Nieder, 2012).

**Figure 2:**
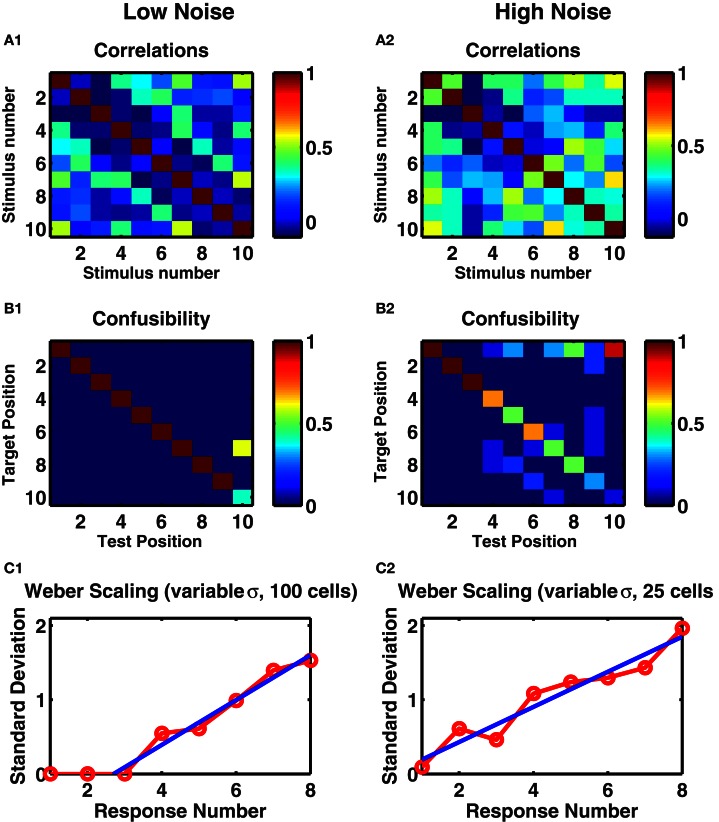
**A randomly connected recurrent network with depressing synapses counts identical stimuli. (A1,A2)** Correlations between mean post-stimulus firing rates of all cell-groups as a function of stimulus number. **(B1,B2)** The confusability matrix indicates the probability of the network activity being most like a given target template following a given number of successive stimuli. Target position corresponds to each of the ten successive stimuli whose mean network activity was evaluated on ten preliminary trials. Recall position denotes each of ten successive stimuli on test trials—following each stimulus, the network activity was measured and compared with target stimuli. Mean of ten trials presented. Color scale: red = 100% correct, green = 50% correct, blue = 0% correct. **(A1,B1)** Internal noise, σ = 0.002. **(A2,B2)** Increased internal noise, σ = 0.005. **(C1,C2)** Standard deviations in the target position as a function of test position. Ten trials of each of ten networks, with different levels of noise, 0.1 < σ < 0.3 in each trial. **(C1)** Network of 100 cell groups. Fitted line to points 3–8 is *y* = 0.30*x* − 0.82. **(C2)** Network of 25 cells. Fitted line to all points *y* = 0.24*x* − 0.04. **(C1,C2)** Straight line fits have higher adjusted *r*-square values than polynomial fits to *y*(*x*) or *x*(*y*), suggesting Weber's Law holds.

When analyzing the complete network response (Figures 1C1,C2) one notices that the overall pattern of activation is distributed: many cell-groups are active following any particular number of stimuli and any one cell-group can be active following multiply different stimuli. However, the activity patterns following particular numbers of stimuli are distinct from each other (Figures 2A1,A2). Indeed, the strongest effect of depression is to decorrelate subsequent stimuli from each other, so the lowest correlation is seen in a band surrounding the diagonal in Figure 2A1. Such an effect can be understood as depression ensuring a group of cells is least likely to be active if it has just been active.

To assess how distinguishable were these different activity patterns from each other, we produced a set of 20 trials by using different instances of temporal noise. We took the mean responses of the first 10 trials to produce “target” responses. We then assessed for each of the next 10 “test” trials, which “target” representation the persistent activity was most similar to. If any two stimuli resulted in the same network response, then the test stimuli would be as often as similar to one as the other, producing a “confusability” of 0.5 to each pair. However, as we see (Figure 2B1), in the low noise case, we found 100% reproducibility of distinct activity patterns for the first 9 of 10 stimulus types. With increased noise, while the first three stimuli remained distinct with 100% reliability, the confusability increased with increasing stimulus count (Figure 2B2).

To quantify the variability in the response, in a separate experiment we selected a different level of noise in each trial used to simulate target responses then test responses. As in the calculation of the confusability matrix, for each stimulus number in a test trial, we treated the network's output as the stimulus number of the target response most correlated with the test response. Across the 10 test trials we calculated the standard deviation of these network outputs. We repeated across 10 different networks to produce the curve in Figure 2C1. With noise in the low range of 0.1 < σ < 0.3, the responses to the first three stimuli are always precisely reproduced, so the variability is zero, but thereafter the standard deviation in the networks' responses increases linearly with stimulus number.

While our standard network comprised 100 excitatory cell-groups (*N*_*E*_ = 100), the qualitative behavior did not depend on this number. With increasing number of cell-groups, the effect of noise was decreased, with an approximate noise-scaling factor of $1/\sqrt{N_{E}}$. Similarly, near identical behavior was produced when the number of cell-groups was reduced, given the appropriate scaling of noise, so that a network with *N*_*E*_ = 25 and σ = 0.001 produced as reliable behavior as a network with *N*_*E*_ = 100 and σ = 0.002. However, when the number of excitatory cell-groups was reduced too much (for example, for *N*_*E*_ < 15) then, with current network parameters and random connections, the network would cycle through a small number of 2–4 discrete states so its ability to count inputs would be severely limited.

The effect of network size can be seen in Figure 2C2, in which we reproduce the analyses leading to Figure 2C1, but with the smaller network of 25 cell-groups. In this case, given the identical range of noise used, more errors occur at any stimulus number, so that even the response to the first stimulus is not completely reliable. The standard deviation of the outputs of 10 such networks is statistically indistinguishable from a straight line through the origin, reproducing Weber's Law of scaling (see Discussion).

### Stimulus duration

**Figure 3:**
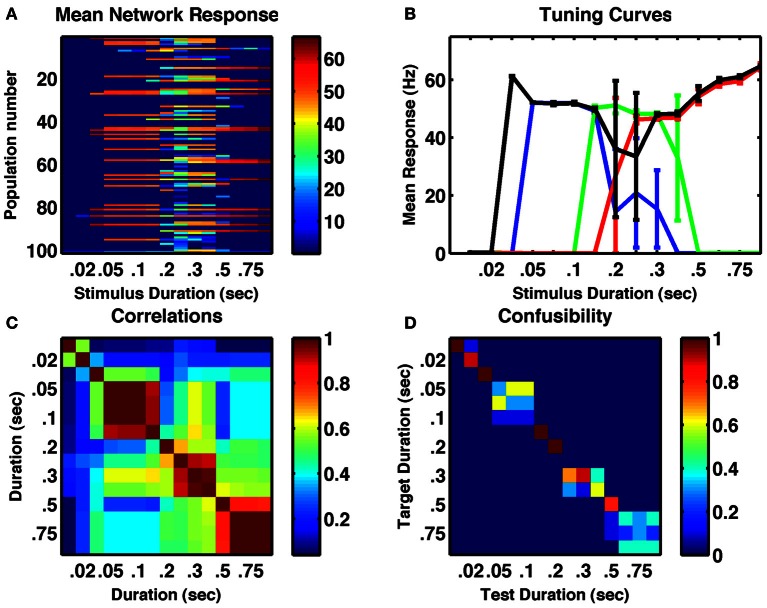
**A randomly connected network with depressing synapses can encode stimulus duration. (A)** Mean response for all cell-groups following a single stimulus as a function of stimulus duration. Color indicates firing rate. **(B)** Responses of four example cell-groups indicate broad tuning. **(C)** Correlation between network firing rates of cell-groups to different stimulus durations. **(D)** The confusability matrix (described in Figure 1) indicates the network can differentiate stimulus duration into seven completely distinct categories. Internal noise, σ = 0.002.

Our network is not an integrator, but relies upon synaptic depression, which has a fixed time constant, to reduce the stability of active states. Therefore, it was not clear whether continuously applied stimuli of fixed durations could have the same effect on network activity as multiple, spaced individual stimuli. To test whether the same network could be responsive to stimulus duration, we reset the network following a range of stimuli of different durations then analyzed the resulting activity. The results in Figure 3, demonstrate the ability of the network to produce a response that is duration-dependent. Seven distinct states of activity are produced in the example network displayed (six if one excludes the unresponsive state following very short stimuli). Interestingly, the tuning curves of individual neurons differ from their tuning to numerosity—they are much broader and more of them are monotonic (Figure 3B).

### Stimulus intensity

**Figure 4:**
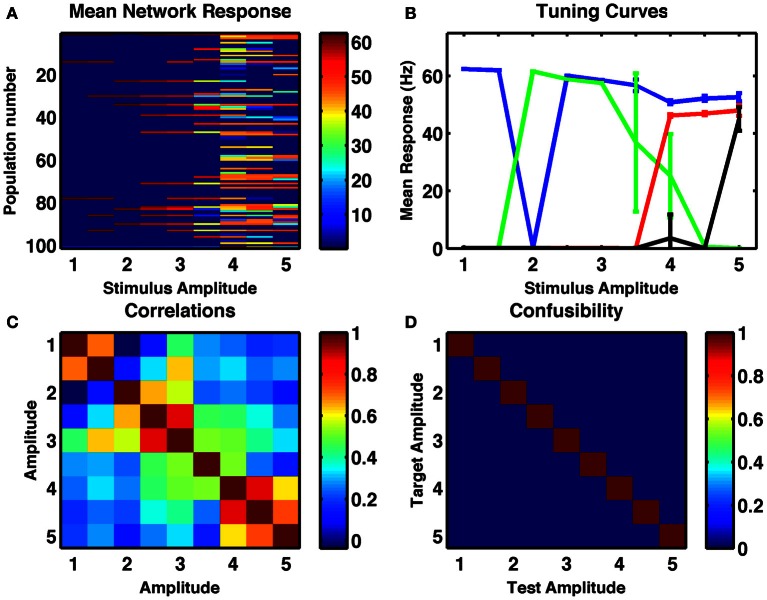
**A randomly connected network with depressing synapses can encode stimulus amplitude. (A)** Mean response for all cell-groups following a single stimulus as a function of stimulus amplitude, ranging in steps of 0.5 from 1 to 5. Color indicates firing rate. **(B)** Responses of four example cell-groups indicate broad tuning to stimulus amplitude. **(C)** Correlation between network firing rates of cell-groups to different stimulus amplitudes. **(D)** The confusability matrix (described in Figure 1) indicates the network can differentiate stimulus amplitude into nine completely distinct categories. Internal noise, σ = 0.002.

We assessed whether the same random network could produce resultant activity that depended on the strength of a fixed duration input current. Results of increasing stimulus strength are similar to those of increased duration in that tuning curves are broader and more monotonic. Interestingly, this is in line with electrophysiological recordings of activities of numerosity-tuned neurons in primates (Nieder and Merten, 2007). Given the broader tuning curves, many pairs of stable activity states were highly correlated (Figure 4C) but in the example shown, all 9 distinct stimulus amplitudes, ranging over a factor of five, were successfully encoded in distinct network states, with 100% reliability (Figure 4D).

### Differentiating number, duration and intensity of stimuli

A perfect integrator would produce a network state-dependent on the product of number, duration and intensity of stimuli. Indeed, one could argue that a drawback to the applicability of the perfect integrator to most sensory tasks is its inability, in the absence of other feedback mechanisms (Machens et al., 2005; Miller and Wang, 2006) to distinguish between number, duration and intensity of stimuli. Moreover, such integrators, as possessed by the head-direction system, or occulomotor system, typically require networks with highly specified architectures and often considerable fine-tuning of parameters. In our formalism, with randomly connected units, the network is robust, because groups of cells are individually bistable. In this manner the network resembles the discrete integrator (Koulakov et al., 2002; Goldman et al., 2003). However, since the connections are random and not tuned to produce the one-dimensional line of stable points typical of an integrator, the network is unlikely to respond to changes in duration, amplitude and number of stimuli in qualitatively the same manner, as does an integrator. Rather, the stable activity on the randomly connected network appears to follow a high-dimensional, distributed representation—different bistable groups can switch on or off with different combinations of other bistable groups, without a systematic order to the switching. Therefore, it is plausible that multiple feature combinations of the stimulus could be separately encoded.

**Figure 5:**
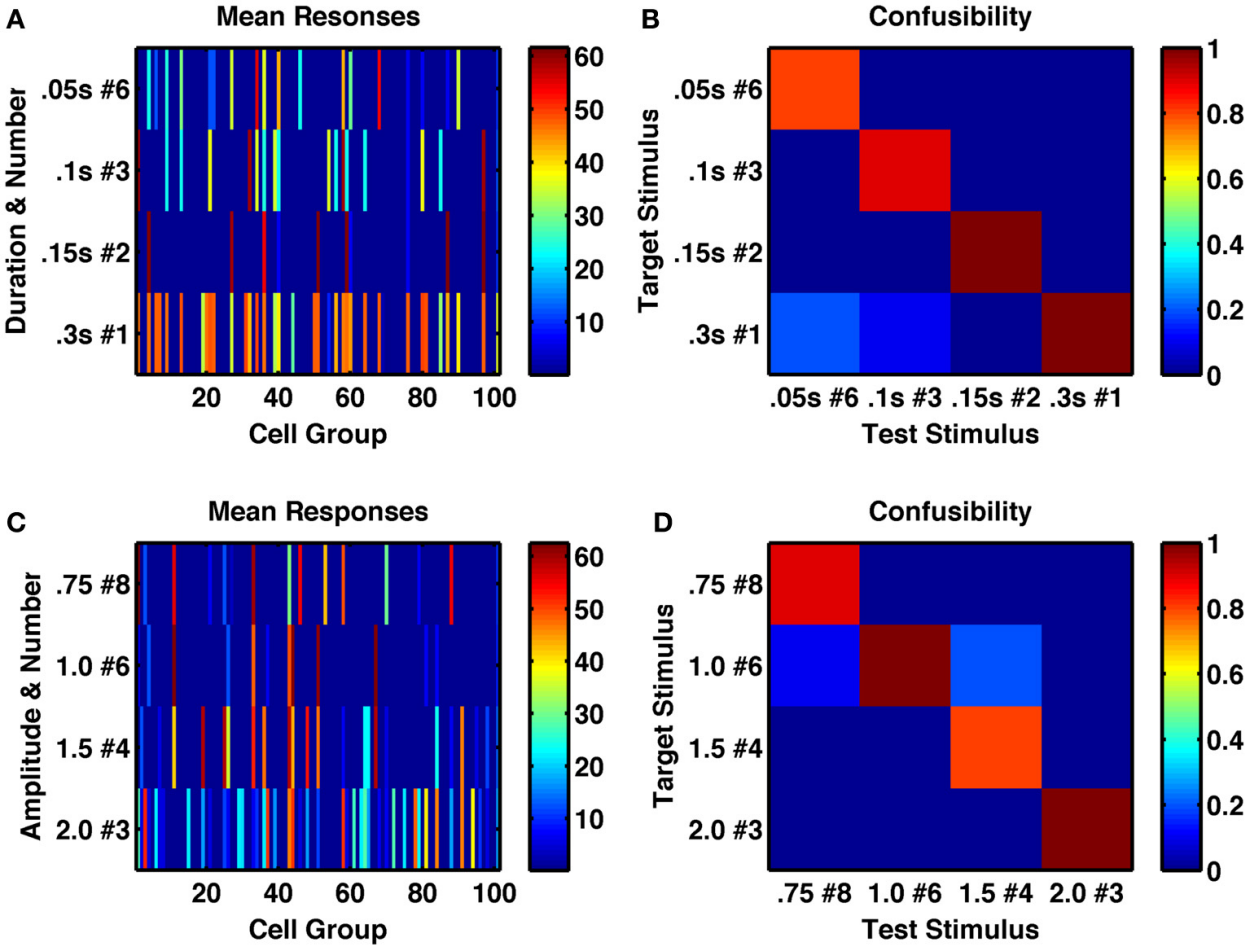
**A randomly connected network with depressing synapses produces distinctive responses to stimulus duration, amplitude and number of repetitions. (A)** Mean network response, with color indicating firing rate of each cell-group, following the 6th of a series of 0.05 s stimulations (row 1), the 3rd of a series of 0.1 s stimulations (row 2), the 2nd of a series of 0.15 s stimulations (row 3) and a single 0.3 s stimulus, such that all stimulus combinations produce 0.3 s of total current (amplitude 1.5, σ = 0.002). **(B)** Confusability matrix between the four types of stimulus combination of **(A)**, indicating the network's response is distinct to each stimulus combination. **(C)** Mean network response as in **(A)** to series of 8, 6 4, and 4 stimuli respectively of different amplitudes 0.75, 1.0, 1.5, and 2.0 (such that the product is constant at 6.0). Each stimulus has duration of 0.25 s, σ = 0.002. **(D)** Confusability matrix between responses to the four stimulus combinations of **(C)** demonstrates the responses are distinct.

**Figure 6:**
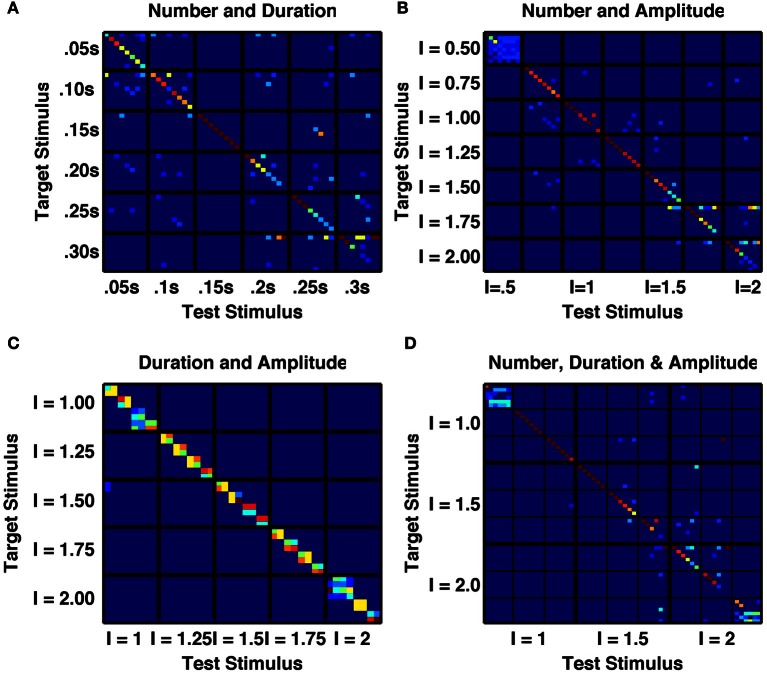
**A randomly connected network with depressing synapses produces distinct responses to multiple stimulus features. (A)** Confusability matrix between the network's activity states following 1–8 transient stimuli of one of six durations from 0.05 s to 0.30 s (48 stimulus combinations in total). 15 combinations are perfectly distinct. **(B)** Confusability matrix between the network's activity states following 1–8 transient stimuli of one of seven amplitudes from 0.5 to 2.0 (56 stimulus combinations in total). 17 combinations are perfectly distinct. **(C)** Confusability matrix between the network's activity states following a single transient stimulus of one of eight durations from 0.05 s to 0.4 s and one of five amplitudes from 1.0 to 2.0 (32 stimulus combinations in total). Although no individual combination is perfectly distinguished from all others, 18 distinct states are apparent, with the majority of states responding to a single amplitude and two durations. **(D)** Confusability matrix between the network's activity states following 1–6 transient stimuli of one of three durations (0.1 s, 0.2 s, 0.3 s) and one of three amplitudes (1.0, 1.5, 2.0). 23 stimulus combinations are perfectly distinguished and over 25 distinct activity states are produced.

To test the ability of the network to represent multiple stimulus features, we first, within a single network, applied trains of transient stimuli of varying durations and constant amplitude. If the network were acting as an integrator, then it would respond to total stimulus time, such that a doubling of the duration combined with halving of the number of stimuli would result in the same network activity. However, we found this not to be the case (Figures 5A,B). Indeed, we analyzed the network's activity following sequences of up to 8 identical transient stimuli, with six different stimulus durations ranging from 0.05 to 0.3 s. We found for the intermediate stimulus duration of 0.15 s that not only was a unique, reliably different activity state produced following each of the eight successive stimuli, but also all 8 states were uniquely produced by that particular stimulus duration and distinct from any states produced by any number of successive stimuli with either longer or shorter durations (Figure 6A).

An integrator would also respond to the product of amplitude and number of stimuli, or amplitude and duration of a single stimulus. However, the randomly coupled network produces distinct responses to trains of a few high-amplitude stimuli and many low-amplitude stimuli, as well as to intermediate combinations when all combinations have the same product of amplitude and number (Figures 5C,D). Moreover, when analyzing the network's activity following sequences of up to eight transient stimuli of constant duration, with seven different amplitudes (in the range 0.5–2.0) we found a very low likelihood for sequences with different amplitudes to be confused with each other and all 8 states following stimuli of intermediate amplitudes to be 90 or 100% correctly identified by both number and amplitude of stimuli (Figure 6B).

Figure 6C further indicates the distinctiveness of network response to stimuli of different amplitudes versus of different durations. Following a single transient stimulus, each of five different stimulus amplitudes in the range 1.0–2.0 produces either 3 or 4 different activity states that depend on stimulus duration. These states are both distinct from each other and distinct from any state produced by another stimulus amplitude (Figure 6C).

We finally produced a 6 × 3 × 3 array of stimuli with any combination of number (*N* = 1 − 6), duration (*T* = 0.1 s, 0.2 or 0.3 s) and intensity (*I* = 1, 2, or 3) of applied current pulses. We assessed how network activity depended on these stimulus combinations. Figure 6D demonstrates that for a large number (27) of these stimulus combinations, the network activity is reliably propelled into a distinct state, unique to that single combination of duration, amplitude and number of stimuli. Since the stimuli are all constant, equal currents to all excitatory cell-groups in the network, the evolution of activity states depends entirely on the random cross-connections between cell-groups and the temporal dynamics of intra-group and inter-group synaptic transmission.

### Networks without depressing synapses

**Figure 7:**
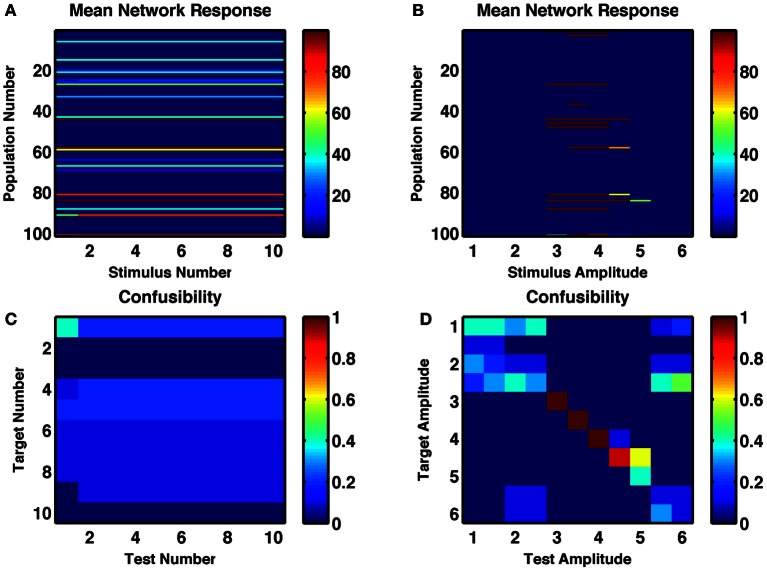
**A random recurrent network without depressing synapses shows no counting behavior and weakly tuned amplitude selectivity. (A,B)** Post-stimulus firing rates of each group of neurons in response to **(A)** repetitions of an optimal-strength stimulus and **(B)** a single stimulus of varying amplitude. **(C,D)** Confusability matrix indicated how distinguishable the network responses are for the successive repetitions of the same stimulus **(C)** or for a single stimulus of different amplitudes **(D)**.

When synaptic depression is removed from these networks—and static release probability is optimally tuned to allow for multiple stable activity states—the counting behavior of the network disappeared (Figures 7A,C). That is, successive stimuli simply reproduced the same state. The number of states produced by different durations and amplitudes of stimuli was reduced from 7–8 to 2–4 (Figures 7B,D). Also, under the same low-noise conditions as the networks shown in Figures 1–6, the reliability of responses to identical stimuli was greatly reduced. In fact, with constant amplitude and varying duration, no states were distinctly produced by a single subset of stimuli.

In summary, it is short-term depression in the recurrent connections of bistable groups that produces itinerancy in the network states. Such itinerancy with consecutive stimuli enables the network to possess a counting behavior and to produce numerosity-tuned cells. The same synaptic depression imparts a preferred stimulus amplitude and duration for activation of a cell-group, increasing the number and reliability of amplitude-specific and duration-specific states.

## Discussion

Bistability relies upon positive feedback, which can arise from cell-intrinsic currents (Hounsgaard et al., 1984; Rinzel, 1985; Booth and Rinzel, 1995) or from network feedback (Kleinfeld et al., 1990; Camperi and Wang, 1998; Wang, 1999, 2001; Koulakov et al., 2002). Synaptic facilitation is a positive feedback mechanism in circuits of reciprocally connected excitatory cells, since the greater the mean firing rate, the greater the effective connection strength, further amplifying the excitatory input beyond that produced by the increased spike rate alone. This property of synaptic facilitation enhances the stability of memory states and renders them more robust to distractors (Itskov et al., 2011). Other forms of positive feedback, such as depolarization-induced suppression of inhibition (DSI), which depends on activity in the post-synaptic cell, can similarly produce robustness in recurrent memory networks (Carter and Wang, 2007).

Conversely, depressing synapses in a self-exciting circuit produce negative feedback, by reducing the effective synaptic strength of the outputs of the most active cells. Such negative feedback reduces the stability of the attractor states produced by positive feedback. This effect has been demonstrated in a system known as the ring attractor, an example of a perfect integrator (Song and Wang, 2005), which in the absence of dynamic synapses can produce a “bump” of population activity in a marginal state. Once the bump has formed at a given location on the “ring” it can remain at that location so form the basis of a spatial memory. However, the stationary “bump” can be rendered unstable by synaptic depression and be replaced by one of two possible moving “bump” states with fixed velocity (York and van Rossum, 2009). Such an effect is similar to that produced by intrinsic adaptation currents within the excitatory neurons of the ring attractor, which result in a pitchfork bifurcation as the single stationary state is replaced by two oppositely directed constant velocity states, whose absolute velocity increases as the underlying conductance increases (Ben-Yishai et al., 1997; Hansel and Sompolinsky, 1998; Laing and Longtin, 2001; Tegnèr et al., 2002).

In the randomly connected circuits that we simulate, synaptic depression in strong recurrent excitatory synapses also has the same effect on these excitatory cells as an adaptation current. Following the initial burst of excitatory input, the dynamic weakening of synaptic strength while vesicles need to be replaced causes a reduction in post-synaptic excitatory input, which affects the post-synaptic cell just as would an activity-dependent intrinsic inhibitory current. Thus, it is possible that synaptic depression could produce similar results to that of an adaptation current in successful models of binocular rivalry based on bistability between groups of neurons (Moreno-Bote et al., 2007; Theodoni et al., 2011).

A randomly connected network of bistable neurons was shown to produce a diversity of neural responses (Rigotti et al., 2010) with neurons possessing mixed selectivity to conjunctions of stimulus features. In that work, different combinations of stimuli or inputs produced the different resulting distributions of stable network activity, allowing for appropriate responses in cognitive tasks. Here, we show that with the addition of depressing synapses, a similar network produces a diversity of responses to different dynamic features of a single stimulus of equal strength to all cells.

The randomly connected network responds differently from neural integrators, whether continuous (Seung, 1996; Miller et al., 2003; Song and Wang, 2005) or discrete (Koulakov et al., 2002; Goldman et al., 2003). For an integrator, increased signal amplitude affects the system in qualitatively the same manner as increased signal duration. The reason for the difference is that integrators are designed to have a one-dimensional sequence of stable fixed points—or a continuous line of fixed points representing a marginal phase (Ben-Yishai et al., 1995), sometimes called a line attractor (Seung, 1996)—whereas the randomly connected network is inherently of high dimensionality (Rigotti et al., 2010). Thus, even when an integrator either inherently (Compte et al., 2000; Song and Wang, 2005) or through its connections to a second output layer (Verguts and Fias, 2004), produces non-monotonic, “peaked” tuning curves, the responses to number, duration and stimulus amplitude are not separable. That is, an integrator's activity following a given number of counts of one stimulus is identical to that following more counts of a weaker stimulus, or of a shorter duration stimulus—of course, in many situations other than counting, such integration is the desired network response (Zhang, 1996; Samsonovich and McNaughton, 1997; Romo et al., 1999; Seung et al., 2000; Song and Wang, 2005).

In many experiments analyzing numerosity coding, both behavioral (Merten and Nieder, 2009) and neural (Nieder and Miller, 2003) responses produce two features suggestive of logarithmic coding. First, errors are skewed, with a longer tail toward stimulus values higher than the stimulus producing peak response. Second, the standard deviation of number estimates—here calculated via the trial-to-trial variability in the network's estimate of stimulus number for each fixed actual number of stimuli—scales linearly with number of stimuli, a scaling known as Weber's Law (Weber, 1851). Our network does not exhibit the observed skew in neural responses, in particular because there is a tendency when errors are made, for the random attractor states visited to be more like the first attractor state (so an incorrect response of “one” is the most common). However, if we incorporate trial-to-trial variability in the level of noise (Figure 2C) then a Weber scaling is observed—errors become more likely, linearly with increasing number. Thus, the information pertaining to the encoded number, as contained within the distributed representation of these networks, degrades in the expected manner, but it is likely a separate “readout” network of cells is needed to produce all the features observed in neural recordings. Such a “readout” network could also combine the different representations of number arising from stimuli of different properties into a single “pure number” representation—that is, it would produce pattern completion after this initial step of pattern separation.

Recent experiments have demonstrated associative forms of short-term plasticity (Brenowitz and Regehr, 2005; Erickson et al., 2010), which is more powerful, since it can be synapse-specific rather than cell-specific, so has greater information carrying capacity. Such associative-STP has been shown to be capable of temporarily coupling together specific pairs of bistable neural groups, so could form the basis for memory of sequences of discrete items (Botvinick and Watanabe, 2007; Miller and Wingfield, 2010).

In summary, we have shown that depression can destabilize discrete activity states and in so doing enables the network activity to change through repetitions of identical stimuli. Therefore, such networks could be of value in providing a basis for counting and for memory of sequences (Botvinick and Plaut, 2006; Botvinick and Watanabe, 2007). Indeed, our ongoing work suggests that memories of discrete sequences could be maintained in a network, which combines such effects of synaptic depression (Figures 1–2) with associative short-term plasticity (Erickson et al., 2010; Miller and Wingfield, 2010).

### Conflict of interest statement

The author declares that the research was conducted in the absence of any commercial or financial relationships that could be construed as a potential conflict of interest.
